# Structural Characterization and Evolutionary Relationship of High-Molecular-Weight Glutenin Subunit Genes in *Roegneria nakaii* and *Roegneria alashanica*

**DOI:** 10.3390/ijms17071115

**Published:** 2016-07-19

**Authors:** Lujun Zhang, Zhixin Li, Renchun Fan, Bo Wei, Xiangqi Zhang

**Affiliations:** 1 State Key Laboratory of Plant Cell and Chromosome Engineering, Institute of Genetics and Developmental Biology, Chinese Academy of Sciences, No. 1 West Beichen Road, Chaoyang District, Beijing 100101, China; zhanglujun000@163.com (L.Z.); lizhixin09@163.com (Z.L.); rcfan@genetics.ac.cn (R.F.); weibo_009@genetics.ac.cn (B.W.); 2 University of Chinese Academy of Sciences, Beijing 100049, China; 3 College of Agriculture, Yangtze University, Jingzhou 434023, Hubei, China

**Keywords:** *Roegneria nakaii*, *Roegneria alashanica*, high-molecular-weight glutenin subunits, gene cloning, variation, phylogenetic analysis

## Abstract

The *Roegneria* of Triticeae is a large genus including about 130 allopolyploid species. Little is known about its high-molecular-weight glutenin subunits (HMW-GSs). Here, we reported six novel HMW-GS genes from *R. nakaii* and *R. alashanica*. Sequencing indicated that *Rny1*, *Rny3*, and *Ray1* possessed intact open reading frames (ORFs), whereas *Rny2*, *Rny4*, and *Ray2* harbored in-frame stop codons. All of the six genes possessed a similar primary structure to known HMW-GS, while showing some unique characteristics. Their coding regions were significantly shorter than *Glu-1* genes in wheat. The amino acid sequences revealed that all of the six genes were intermediate towards the y-type. The phylogenetic analysis showed that the HMW-GSs from species with St, StY, or StH genome(s) clustered in an independent clade, varying from the typical x- and y-type clusters. Thus, the *Glu-1* locus in *R. nakaii* and *R. alashanica* is a very primitive glutenin locus across evolution. The six genes were phylogenetically split into two groups clustered to different clades, respectively, each of the two clades included the HMW-GSs from species with St (diploid and tetraploid species), StY, and StH genomes. Hence, it is concluded that the six *Roegneria* HMW-GS genes are from two St genomes undergoing slight differentiation.

## 1. Introduction

High-molecular-weight glutenin subunits (HMW-GSs) are the major seed storage proteins in the endosperm of wheat and its related species. In common wheat (*Triticum aestivum* L., AABBDD), HMW-GSs are the critical determinants of wheat processing quality as their composition affects the viscoelastic properties of dough [1,2]. It is well-known that the common wheat HMW-GSs are encoded by three *Glu-1* loci, namely *Glu-A1*, *Glu-B1*, and *Glu-D1*, located on the long arm of the group 1 homeologous chromosomes [3,4], and each locus comprises of two tightly linked genes, encoding one x-type and one y-type subunit, respectively [5,6].

Since the 1980s, a series of HMW-GS genes have been isolated from wheat cultivars, and their structural characteristics have been molecularly documented in great detail [7,8,9,10,11]. Orthologous HMW-GSs have also been found, and some of their coding genes have been isolated from several Triticeae grasses, such as *Aegilops*, *Australopyrum*, *Crithopsis*, *Elymus*, *Eremopyrum*, *Leymus*, *Psathyrostachys*, *Pseudoroegneria*, *Secale*, and *Thinopyrum* genera [12,13,14,15,16,17,18,19,20,21,22,23]. A large number of studies demonstrated that wheat HMW-GSs shared the same primary structure with those of the related species, including the signal peptide (which is removed in the mature HMW-GS), highly-conserved N-terminal and C-terminal domains, and a central repetitive region composed mainly of tri-, hexa-, and nonapeptide motifs [4,24]. The extensive investigation indicated that the allelic variation of the *Glu-1* loci in wheat-related species was very abundant, and those novel variants could also potentially improve the quality of the wheat variety.

*Roegneria* C. Koch is based on *Roegneria* *caucasica* C. Koch. *Roegneria* is a vast and widely-distributed genus in the tribe Triticeae (Poaceae), including about 130 species globally, and primarily distributed in Asia, while only a few of species grow in Southern Europe and North America [25,26]. All of the species of *Roegneria* are allopolyploids with the St and Y genomes and two ploidy types occur naturally according to the cytogenetic taxon, i.e., tetraploid (2n = 4x = 28, StStYY) and hexaploid (2n = 6x = 42, StStStStYY) [26]. Some of the collections possess valuable traits for wheat improvement, such as tolerance to drought, low temperature, salinity stresses, and resistance to diseases [27,28,29].

Hitherto, little information is available on the *Roegneria* HMW-GSs and their coding genes. Herein, we report six novel alleles of HMW-GS genes from two tetraploid *Roegneria* species (StStYY) native to China, *R. nakaii* Kitag and *R. alashanica* Keng, and discuss their structural characteristics and evolutionary status.

## 2. Results

### 2.1. The Composition of HMW-GSs in R. nakaii and R. alashanica

**Figure 1:**
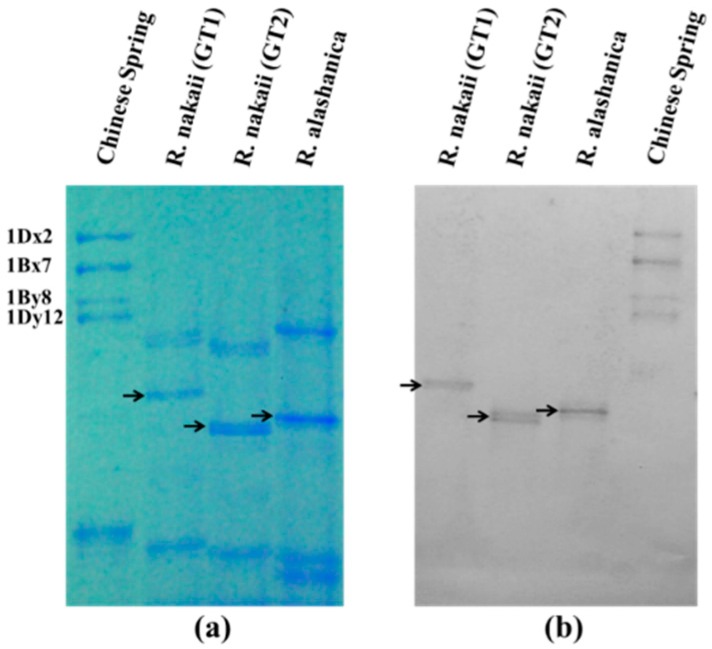
Sodium dodecyl sulfate-polyacrylamide gel electrophoresis (SDS-PAGE) (**a**) and Western blotting (**b**) patterns of the high-molecular-weight glutenin subunits (HMW-GSs) from *R. nakaii* and *R. alashanica*. The HMW-GSs in *Roegneria* species are indicated by arrows. The HMW-GSs (1Dx2, 1Bx7, 1By8, and 1Dy12) of common wheat variety Chinese Spring were used as controls.

The HMW-GSs of *R. nakaii* and *R. alashanica* were analyzed by sodium dodecyl sulfate-polyacrylamide gel electrophoresis (SDS-PAGE) (Figure 1a) and Western blotting (Figure 1b). The patterns showed that only one subunit was expressed in each examined seed of the two *Roegneria* species, and a total of two subunits with different sizes in *R. nakaii* (in GT1 and GT2, respectively) and one subunit in *R. alashanica* were identified among the detected seeds.

A striking feature of these *Roegneria* HMW-GSs was their considerably faster electrophoretic mobilities compared to the subunits from common wheat. Additionally, the mobility of the largest subunit (in GT1 of *R. nakaii*) was faster than that of the 1Dy12 subunit of Chinese Spring, and these were distributed in the same region wherein low-molecular-weight glutenin subunits (LMW-GSs) or ω-gliadins appeared (Figure 1). In addition, there were some other protein bands with slower electrophoretic mobility on SDS-PAGE than those of the HMW-GS homologs which were not recognized by the antibody. We speculated that those protein bands might be gliadin homologs.

### 2.2. Cloning and Sequence Analyzing of HMW-GS Genes from *R. nakaii* and *R. alashanica*

**Figure 2:**
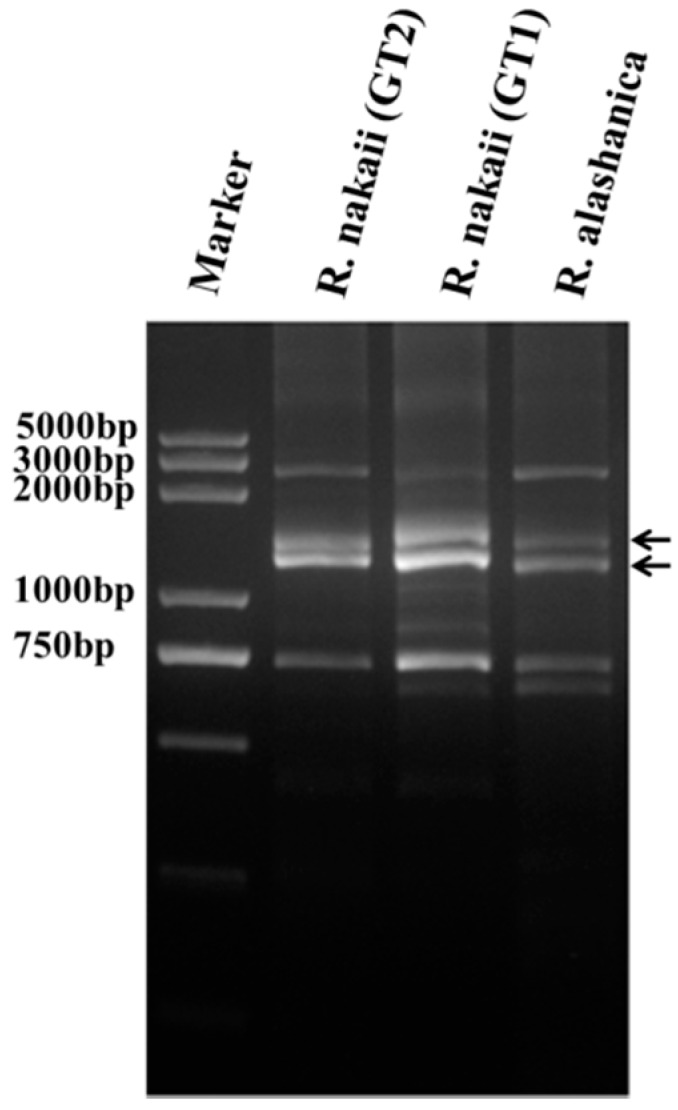
Electrophoretic profiles of PCR products corresponding to the complete coding sequences of HMW-GS genes from *R. nakaii* and *R. alashanica*. The target bands are indicated by arrows.

Since the HMW-GS genes do not harbor introns, we thus used a pair of degenerate PCR primers, P1/P2, to amplify the coding sequence of HMW-GS from the genomic DNA of *R. nakaii* (GT1 and GT2) and *R. alashanica* [9]. The amplicons displayed 4–5 bands for each genotype on agarose gel electrophoresis (Figure 2). The PCR fragments between 1000 and 2000 bp, which were HMW-GS genes, were excised, purified and sequenced. Overall, six HMW-GS genes were obtained from the two *Roegneria* species. Among them, four genes (named *Rny1–Rny4*) were derived from *R. nakaii* (*Rny1* and *Rny2* from GT2, and *Rny3* and *Rny4* from GT1) and two genes (named *Ray1* and *Ray2*) from *R. alashanica*.

Sequence analysis revealed that the lengths of *Rny1*, *Rny2*, *Rny3*, *Rny4*, *Ray1*, and *Ray2* genes were 1371 bp, 1253 bp, 1452 bp, 1280 bp, 1389 bp, and 1217 bp (including the tandem stop codons), respectively. *Rny1*, *Rny3*, and *Ray1* possessed intact open reading frames (ORF), while *Rny2*, *Rny4*, and *Ray2* contained one (*Rny4*) or two (*Rny2* and *Ray2*) in-frame stop codons at the central repetitive domain. Moreover, there was a single base deletion at the C-terminal of each of these three genes (1223rd in *Rny2*, 1250th in *Rny4*, and 1187th in *Ray2*) which led to frame shift. BLAST analysis of the entire sequences indicated that the six novel genes were highly homologous to the wheat HMW-GS genes, and were similar to the y-type HMW-GS genes. The sequences of the six HMW-GS genes from *Roegneria* species have been deposited into GenBank [30] with the accession numbers KP121399 for *Ray2*, KP121400 for *Ray1*, KP121401 for *Rny2*, KP121402 for *Rny1*, KP121403 for *Rny4*, and KP121404 for *Rny3* (Table 1).

**Figure 3:**
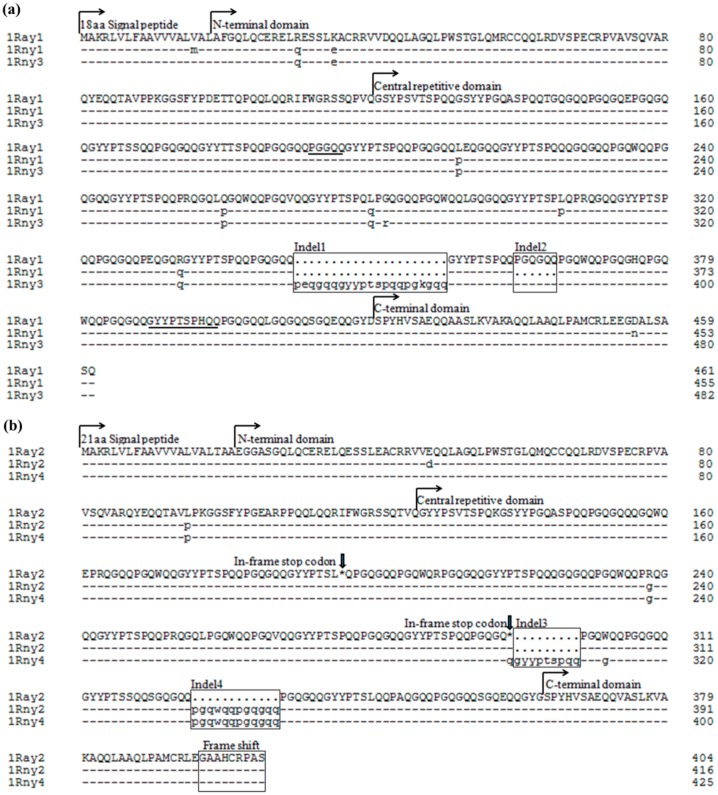
Comparison of the deduced amino acid sequences of Rny1, Rny3, and Ray1 (**a**); and Rny2, Rny4, and Ray2 (**b**). The irregular repeat motifs are underlined, indels and frame shift region are boxed, and in-frame stop codons are marked by solid arrows.

Multiple alignment analyses indicated that *Rny1*, *Rny3*, and *Ray1* were highly identical at the nucleotide sequence level. Sixteen single nucleotide polymorphisms (SNPs) and two DNA fragment indels (63 bp indel was named as Indel1 and 18 bp indel was named as Indel2) were found among them. Concretely, six SNPs and two indels (Indel1 and Indel2) between *Rny1* and *Rny3*, fifteen SNPs and Indel2 between *Rny1* and *Ray1*, and eleven SNPs together with Indel1 between *Rny3* and *Ray1* were found (Figure S1a and Figure 3a). *Rny2*, *Rny4*, and *Ray2* were also highly identical to each other, with ten SNPs and two DNA fragment indels (27 bp indel was named as Indel3 and 36 bp indel was named as Indel4) occurring among them. Eight SNPs and Indel3, four SNPs and Indel4, and eight SNPs together with Indel3 and Indel4 were detected between genes *Rny2* and *Rny4*, *Rny2* and *Ray2*, and *Rny4* and *Ray2*, respectively (Figure S1b and Figure 3b).

### 2.3. Characterization and Comparative Analysis of the Roegneria HMW-GSs

Analyses of the deduced primary structures of the six novel *Roegneria* subunits revealed that they shared the same essential structure with the HMW-GSs characterized previously, composed of the signal peptide, N-terminal domain, central repetitive domain, and C-terminal domain (Figure 3, Table 1).

**Figure 4:**
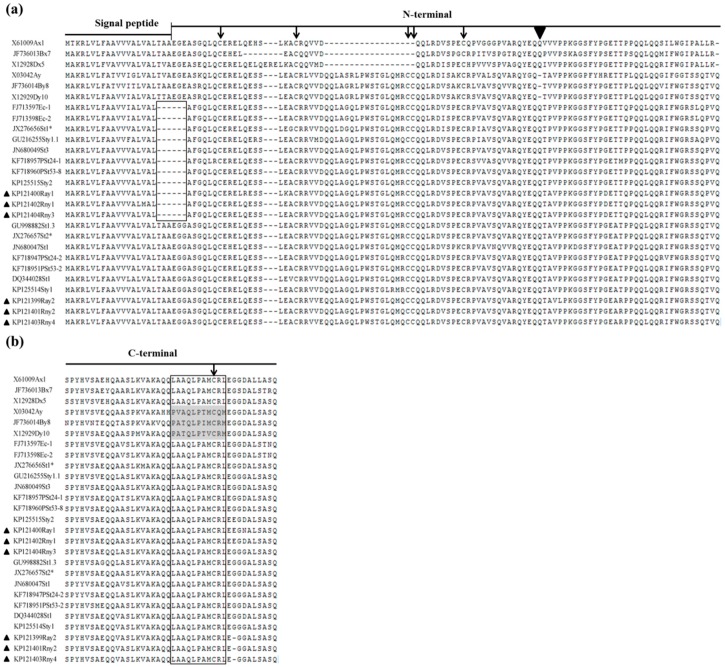
Sequence alignment of the six novel *Roegneria* HMW-GS genes, along with the other representative HMW-GS genes. (**a**) Signal peptides and N-terminal domains; (**b**) C-terminal domains. The six *Roegneria* HMW-GS genes are indicated by solid triangles on the left. The deleted hexapeptide crossing signal peptide and N-terminal domain in some subunits, and an undecapeptide (LAAQLPAMCRL) in the C-terminal associated with x-type subunits in wheat are marked by boxes, and the different undecapeptides of y-type subunits in wheat are shadowed. The conserved cysteine residues in the N- and C-terminal domains are indicated by arrows. The extra glutamine residue conserved in the N-terminal of all x-type and some y-type subunits is marked by an inverted solid triangle on top of the subfigure (**a**).

The subunits Rny2, Rny4, and Ray2 had a common signal peptide sequence composed of 21 residues. However, in subunits Rny1, Rny3 and Ray1, the signal peptide had three deleted residues and consisted of 18 residues (Figure 3 and Figure 4a). The N-terminal domain of Rny2, Rny4, and Ray2 contained 105 residues, while that of Rny1, Rny3, and Ray1 encompassed 102 residues. The shorter signal peptide and the N-terminal domain of Rny1, Rny3, and Ray1 were attributed to a hexapeptide deletion (boxed in Figure 4a) crossing the signal peptide and N-terminal domain. Compared to the wheat HMW-GSs, the N-terminal domains of the six *Roegneria* subunits contained an extra glutamine residue (marked by an inverted solid triangle in Figure 4a), which was absent in the y-type subunits of common wheat, but present in all of the known x-type subunits. The N-terminal regions of all the six subunits possessed five conservative cysteine residues, located exactly parallel to those of the typical y-type subunits in wheat. The N-terminal domains of Rny1, Rny3, and Ray1 ended with a hexapeptide, SSQPVQ, which was found only in a few HMW-GSs derived from the St genome, while Rny2, Rny4, and Ray2 ended with the y-type subunit specific hexapeptide SSQTVQ (Figure 4a).

The conserved C-terminal region of the subunits Rny1, Rny3, and Ray1 was normally composed of 42 amino acid residues, while that of Rny2, Rny4, and Ray2 showed a single base deletion on the 34th triplet codon, which led to the frame shift of the ORF. The undecapeptide LAAQLPAMCRL associated with x-type subunits was also found in the C-terminal of all the six *Roegneria* subunits (Figure 4b).

The central repetitive domains of the six novel subunits from *Roegneria* species were similar to those of the representative y-type subunits of wheat in their structures of tandem and interspersed repeats based on hexapeptide and nonapeptide. Despite the conserved structural characteristics, the following particular aspects were observed in these subunits. (1) The central repetitive domain lengths (Table 1, 238–320 residues) were obviously shorter than those of the representative y-type HMW-GSs in common wheat. The shorter central repetitive region was a radical cause leading to smaller molecular masses and, thus, accelerated SDS-PAGE mobility (Figure 1); (2) cysteine residue did not occur near the C-terminus of the repetitive region of all the six *Roegneria* subunits, which was distinct from the typical y-type subunits of common wheat, especially that encoded by the *Glu1-B1* and *Glu1-D1* loci, but similar to the typical x-type subunits; (3) the two irregular motifs, pentapeptide PGGQQ and decapeptide GYYPTSPHQQ, were found in the central repetitive region of Rny1, Rny3, and Ray1 (Figure 3a), and they could be inferred as the mutants of typical hexapeptide (PGQGQQ) and nonapeptide (GYYPTSPQQ); (4) one or two stop codon(s) appeared in the central repetitive region of Rny2 (two), Rny4 (one), and Ray2 (two) (Figure 3b). Hence, these three were pseudogenes without protein products.

Amino acid sequence comparison of the repetitive regions of the six *Roegneria* subunits found two amino acid fragment indels, the 21-residue fragment PEQGQQGYYPTSPQQPGKGQQ (corresponding to Indel1, Figure S1a) and hexapeptide fragment PGQGQQ (corresponding to Indel2, Figure S1a) among the subunits of Rny1, Rny3, and Ray1. In addition, two amino acid substitutions occurred in the repetitive regions of Rny1 and Rny3, and five between Rny1 and Ray1 and Rny3 and Ray1, respectively (Figure 3a). Additionally, two amino acid fragment indels, nonapeptide QGYYPTSPQ (corresponding to Indel3, Figure S1b) and dodecapeptide PGQGQQPGQGQQ (corresponding to Indel4, Figure S1b) were found among Rny2, Rny4, and Ray2, whereas two, one, and three amino acid substitutions occurred in the repetitive region of Rny2 and Rny4, Rny2 and Ray2, and Rny4 and Ray2, respectively (Figure 3b).

### 2.4. Heterologous Expression of the Roegneria HMW-GS Genes in Escherichia coli

**Figure 5:**
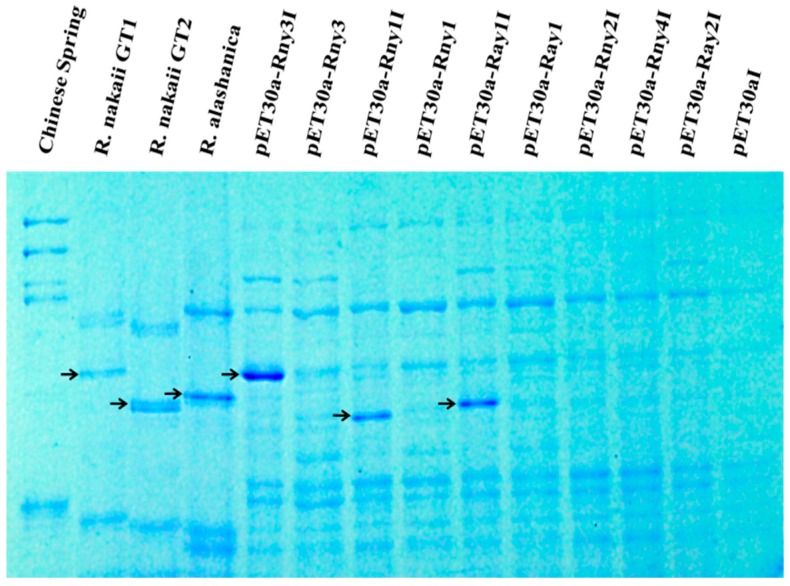
Heterologous expression of the HMW-GS genes from *R. nakaii* and *R*. *alashanica* in *E. coli*. The electrophoresis patterns showed the three isopropyl β-d-1-thiogalactopyranoside (IPTG)-induced *Roegneria* HMW-GSs in *E. coli*, Rny1, Rny3, and Ray1 had the same mobility with those of HMW-GSs extracted from *Roegneria* seeds. The three pseudogenes, *Rny2*, *Rny4* and *Ray2*, did not express protein under the IPTG-induced condition. The HMW-GSs extracted from *Roegneria* seeds (lanes *R. nakaii* GT1, GT2, and *R. alashanica*) and expressed in *E. coli* (lanes pET30a-*Rny3*I, pET30a-*Rny1*I, and pET30a-*Ray1*I) are marked by arrows. The Chinese Spring HMW-GSs (1Dx2, 1Dy12, 1Bx7, 1By8) extracted from seeds were used as controls.

To confirm the expression of the *Roegneria* HMW-GS genes, they were cloned and overexpressed in *Escherichia coli*. The nucleotide sequences encoding the signal peptides in the ORF sequences were removed for the comparable electrophoretic mobilities of the mature proteins. SDS-PAGE analysis of the proteins extracted from the transformed *E. coli* cells showed that the three genes *Rny1*, *Rny3*, and *Ray1* successfully expressed the specific protein subunits and that they matched with their counterparts derived from *R. nakaii* and *R. alashanica* endosperm proteins, respectively (Figure 5). In contrast, no protein was expressed in the transformants of the three constructs pET30a-*Rny2*, pET30a-*Rny4*, and pET30a-*Ray2* (Figure 5), which established them as non-expressional pseudogenes.

### 2.5. Phylogenetic Relationships of the New Roegneria HMW-GSs with Orthologous Subunits from Other Species

**Figure 6:**
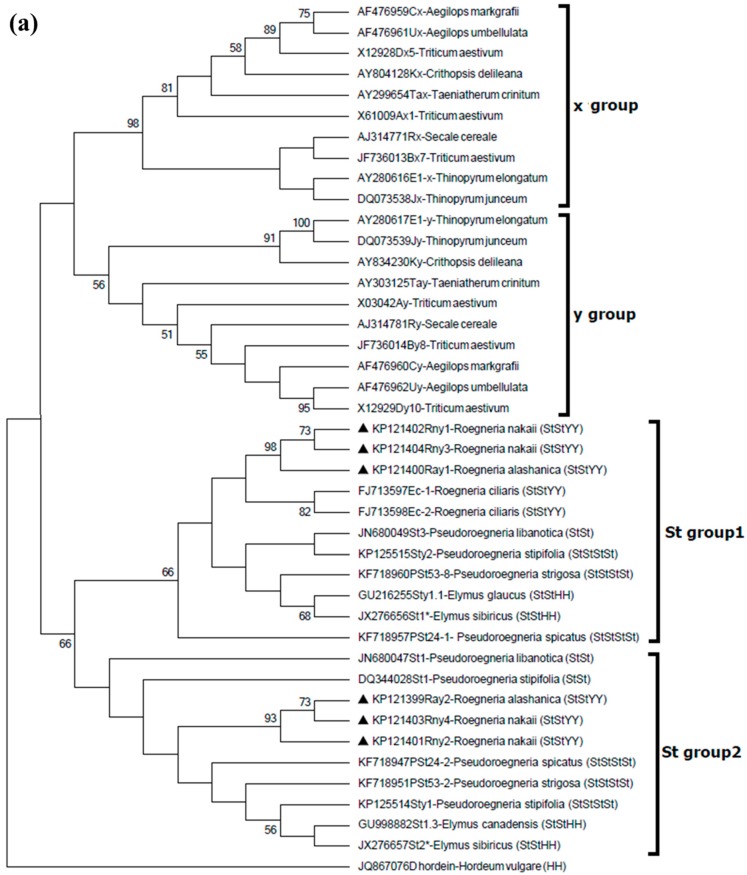

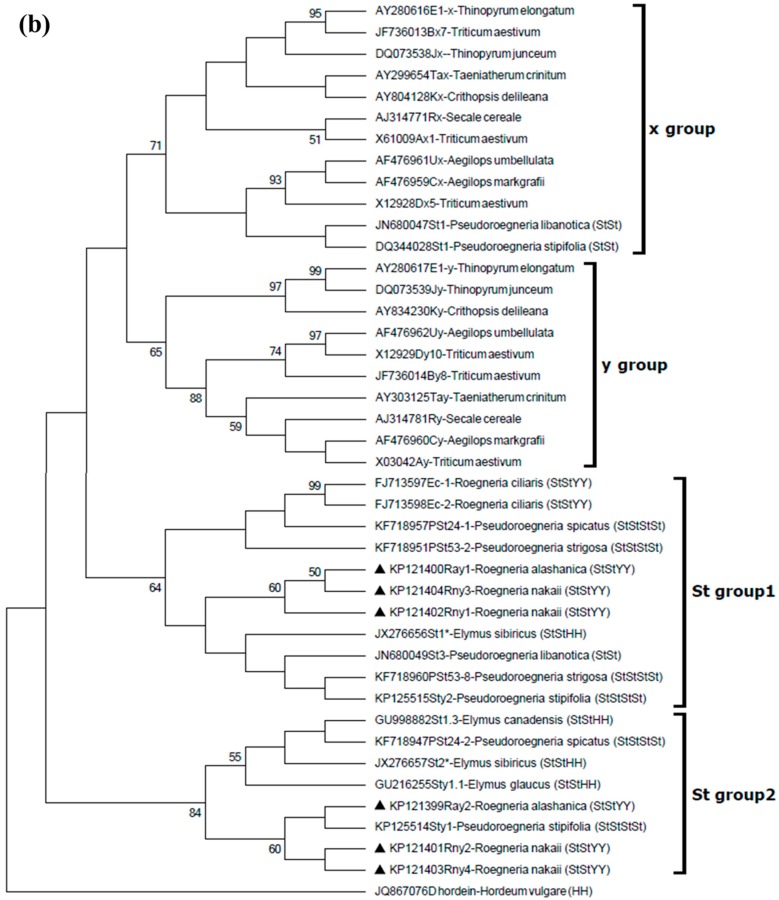
Phylogenetic trees of HMW-GSs from *R. nakaii* and *R. alashanica* and their orthologous subunits from wheat and other related species. The phylogenetic trees were constructed based on the amino acid sequences of N-terminal with the first three motifs of the central repetitive domain (**a**) and the C-terminal with the last six motifs of the central repetitive domain (**b**) using maximum likelihood method. Bootstrap values ≥50% estimated based on 500 replications are shown above the branches. The six novel *Roegneria* subunits are marked by solid triangles.

To investigate the evolutionary relationships among the six *Roegneria* HMW-GSs characterized in this study and some of the representative HMW-GS in wheat and related species with different genomes (E, J, R, C, U, Ta, and K), two maximum likelihood (ML) phylogenetic trees were constructed. ML was based on the amino acid sequences of the N-terminal with the first three motifs of the central repetitive domain and the C-terminal plus the last six motifs of the central repetitive domain of these subunits, respectively. The two phylogenetic trees exhibited very small differences in the topological structure and support values for the branches (Figure 6).

According to the topology of the phylogenetic tree constructed based on N-terminal plus motifs of the central repetitive domain, the HMW-GSs were categorized into four groups. All of the x-type HMW-GSs formed the first group (x group), and the y-type HMW-GSs, except the subunits derived from the St genome, formed the second group (y group). The subunits derived from the St genome were divided into two groups (St group 1 and St group 2), which were distinct with the x and y groups (Figure 6a). Similarly, the six novel *Roegneria* HMW-GS genes were also split into two small groups, which were paralleled by the two St groups. Rny1, Rny3, and Ray1 formed a small clade, and they belonged to the St group 1, together with the other eight HMW-GSs derived from *R. ciliaris* (Ec-1, Ec-2), *Pseudoroegneria libanotica* (St3), *Pd. spicatus* (PSt24-1), *Pd. stipifolia* (Sty2), *Pd. strigosa* (PSt53-8), *Elymus glaucus* (Sty1.1), and *E. sibiricus* (St1*), respectively. The other three subunits Rny2, Rny4, and Ray2 were also closely associated with each other forming a small group, and they clustered in another separate clade (St group 2) together with St1 (*Pd. libanotica*), PSt24-2 (*Pd. spicatus*), St1 (*Pd. stipifolia*), Sty1 (*Pd. stipifolia*), PSt53-2 (*Pd. strigosa*), St1.3 (*E. canadensis*), and St2* (*E. sibiricus*) (Figure 6a).

In accordance with the phylogenetic tree constructed based on the C-terminal plus motifs of central repeat domain, the HMW-GSs analyzed in this study could also be classified into four groups. Although small differences in the topological structure and support values for the clades were found between the phylogenetic trees based on the C- and N-terminals, they were highly similar with each other. Rny1, Rny3, and Ray1 together with the other eight subunits derived from the St genome composed the St group 1; Rny2, Rny4, Ray2 and other five St subunits belonged to St group 2 (Figure 6b). Among the subunits from the St genome, the subunit St1 (accession No. JN680047) from *Pd. libanotica* and Glu-1St1 (accession No. DQ344028) from *Pd. stipifolia* had their N-terminals clustered together with St subunits (St group 2), but their C-terminals were more similar to those of the typical x-type subunit (Figure 6b, x group).

## 3. Discussion

Recently, a number of HMW-GS genes from wheat-related grasses were molecularly investigated. However, until now, the HMW-GS of the *Roegneria* genus were rarely studied. In the present study, we isolated six novel HMW-GS genes from two tetraploid *Roegneria* species endemic to China, *R. nakaii* and *R. alashanica*. Among them, the genes *Rny1*, *Rny3*, and *Ray1* possessed intact ORFs, and *Rny2*, *Rny4*, and *Ray2* were pseudogenes containing in-frame stop codon(s). The analyses of the coding regions and the corresponding amino acid sequences indicated that these *Roegneria* HMW-GSs shared the same essential molecular structures with those of the common wheat and other related species. Each was composed of four domains, a signal peptide (which would be removed from the matured subunit), conserved N- and C-terminals, and a longer central repetitive region. These novel *Roegneria* subunit genes expanded the HMW-GS gene family and laid the foundation for investigating their perspective on wheat improvement and evolution of the *Glu-1* locus in the tribe Triticeae.

The novel *Roegneria* HMW-GS genes also showed specific properties in the sequence structures. The DNA sequence analyses indicated that the six genes (1217–1452 bp, Table 1) were notably shorter than the common HMW-GS genes in wheat, which ranged from 1800 to 2500 bp [31]. For the three genes with intact ORFs (*Rny1*, *Rny3*, and *Ray1*), this feature was consistent with their protein productions with fast electrophoretic mobility (Figure 1). The deduced amino acid sequences from the six *Roegneria* HMW-GS genes revealed that the smaller molecular size was mainly due to the fewer repeat motifs in the central repetitive domain (Figure 4). The smaller HMW-GS subunits have also been found in some other wheat related genera, such as *Pseudoroegneria* [14,22,32], *Elytrigia* (=*Agropyron*) [15,19], and *Elymus* [16], thereby suggesting that smaller HMW-GS is common in wheat-related species, but little is known about its evolutionary significance and perspective on wheat improvement and, thus, merit further studies.

The analyses of the deduced amino acid sequences from the six *Roegneria* HMW-GS genes showed that these subunits possessed some essential structural features of the typical y-type HMW-GSs, such as N-terminal composed of 105 (subunits Rny2, Rny4, and Ray2) or 102 (subunits Rny1, Rny3, and Ray1) amino acid residues, five conserved cysteine residues in N-terminal sequences (Figure 4), and no tripeptide GQQ (x-type subunit-specific) in the central repetitive domains (Figure 3). Nevertheless, these *Roegneria* HMW-GSs also showed some structural characteristics of the x-type subunits, such as the N-terminal domains of all the six HMW-GSs possessing an additional glutamine residue (a solid triangle indicated in Figure 4a), which was typically presented in the x-type subunits of wheat and some y-type subunits derived from the wild species with E, F, K, O, P, Q, Ta, V, and W genomes [13,17,18,19], and these y-type subunits were deemed to evolutionary precede those without this glutamine residue [18,32]. The C-terminal of all of the six *Roegneria* HMW-GSs demonstrated a distinctive undecapeptide, LAAQLPAMCRL (Figure 4b), which is associated with x-type subunits. In addition, in the phylogenetic trees constructed based on the N- and C-terminal amino acid sequences, these *Roegneria* HMW-GSs were closely associated with the subunits derived from species with the St genome (St, StY, or StH). All of these subunits formed an independent clade, which had a long genetic distance with the typical x- and y-type subunits derived from A, B, and D genomes of wheat and E, J, R, C, U, Ta, and K genomes of the related species involved in this study (Figure 6).

As per the above characteristics, these six *Roegneria* HMW-GS genes are not a typical y-type gene, and should rather be classed into an intermediate type inclining towards the y-type. Some atypical or intermediate type HMW-GSs have also been found in other Triticeae species, such as *Agropyron cristatum* [33], *Elymus* *glaucus* [16], and several *Pseudoroegneria* species [14,22], suggesting the atypical HMW-GS is universally existent in the wild species of the tribe Triticeae, and plausibly represents an evolution status of the *Glu-1* locus.

Shewry et al. proposed that the x- and y-type HMW-GS genes derived from a common ancestral gene similar to the y-type genes [24]. However, no x-type subunit genes have been identified from the *Glu-St1* locus until now [14,22,32]. It was speculated that the St genome differentiated from the modern-day Triticeae genomes earlier than the differentiation between x- and y-type genes [22]. The phylogenetic analysis in this study was in agreement with the hypothesis. It further showed that the HMW-GSs from the species with St, StY, or StH genome(s) clearly clustered as an independent clade that was genetically distal from the typical x- and y-type clusters composed of HMW-GSs from wheat and other related species with seven different genomes (Figure 6). Thus, based on the evidence, we infer that the *Glu-1* locus in *R. nakaii* and *R. alashanica* is a very primitive glutenin locus across evolution.

*R. nakaii* and *R. alashanica* have the same genome compositions, StStYY [26]. Phylogenetic analysis showed that the six *Roegneria* HMW-GS genes were split into two groups clustered to different clades, respectively (Figure 6, St group 1 and St group 2), suggesting that maybe they were derived from two separate genomes. However, it was apparent from the phylogenetic trees that each one of the two clades (St group 1 or St group 2) included the HMW-GSs from species with only St (diploid and tetraploid species), StY, and StH genomes. Hence, we speculate that two group HMW-GS genes in *Roegneria* are all derived from the St genome rather than Y. Nevertheless, the two genomes of the two groups of *Roegneria* HMW-GS genes have undergone slight differentiation.

## 4. Materials and Methods

### 4.1. Plant Materials

Two tetraploid *Roegneria* species, *R. nakaii* Kitag (No. Z1874) and *R. alashanica* Keng (No. Z1081), were used in this study, and their mature seeds were kindly provided by Prof. Lihui Li (Institute of Crop Sciences, Chinese Academy of Agricultural Sciences, Beijing, China). The common wheat cultivar Chinese Spring was used as a control in SDS-PAGE and Western blotting experiments, and its mature seeds were from our laboratory collections.

### 4.2. SDS-PAGE

The HMW-GSs were extracted from mature seeds of *R. nakaii* and *R. alashanica* and separated by SDS-PAGE as described previously [34]. The HMW-GSs from the common wheat cultivar Chinese Spring (Null, 1Bx7 + 1By8, 1Dx2 + 1Dy12) were used as standards. At least ten individual seeds were examined to ascertain the composition of HMW-GS in the given accessions.

### 4.3. Western Blotting

The proteins on the polyacrylamide gel were transferred to an Amersham Hybond-P PVDF Membrane (GE Healthcare, Uppsala, Sweden) by electroblotting at 90 V for 3 h. The membrane was washed for 10 min with TBST buffer (0.01 M Tris-HCl, 0.15 M NaCl pH 7.4, and 0.05% Tween 20) and blocked in TBSTM (5% skim milk in TBST) for 2 h. After three subsequent washes, the membrane was incubated in TBSTM with a 1:7000 dilution of the anti-R2 polyclonal antibody [35] for 2 h, followed by horseradish peroxidase (HRP)-labelled goat anti-rabbit IgG (Beyotime, Shanghai, China) for 2 h. Consecutively, the membrane was washed three times and then incubated in precipitating TMB (3,3′,5,5′-tetramethylbenzidine) Substrate Solution (Beyotime) for 10–30 min. The reaction was stopped by washing the membrane with distilled water.

### 4.4. Cloning and Sequencing of the Roegneria HMW-GS Genes

Genomic DNA was extracted from fresh leaves of seedlings using the cetyltrimethylammonium bromide (CTAB) method described by Webb and Knapp [36]. A pair of degenerate primers, P1: 5′-ATGGCTAAGCGGC/TTA/GGTCCTCTTTG-3′ and P2: 5′-CTATCACTGGCTG/AGCCGACAATGCG-3′ [9], were used to amplify the ORFs of HMW-GS genes from *R. nakaii* and *R. alashanica* using the high-fidelity PrimeSTAR HS DNA Polymerase with GC buffer II (TaKaRa, Dalian, China) to avoid PCR errors. The PCR was as follows: denaturation for 3 min at 98 °C; followed by 30 cycles of 20 s at 98 °C, 30 s at 69 °C, and 3 min at 72 °C; extended finally for 10 min at 72 °C. The amplicons were separated on 2.0% agarose gel, and the anticipated band was excised and purified using a TIANgel Midi Purification Kit (TIANGEN, Beijing, China). The purified DNA fragments were cloned into pZeroBack/Blunt vector (TIANGEN). The recombinant plasmids were transformed into *Escherichia coli* DH5α competent cells (TIANGEN). Nucleotide sequencing was performed commercially (Beijing Genomics Institute, Beijing, China) and the final sequence for each HMW-GS gene was determined from the sequencing results of several independent clones.

### 4.5. Heterologous Expression of Cloned HMW-GS Genes in E. coli

The six HMW-GS genes, excluding the sequences of the signal peptides, were amplified by PCR using the following primers: P3-1: 5′-GGAATTCCATATGGCCTTTGGGCAACTACAGTGTGAG-3′ for *Ray1*, *Rny1*, and *Rny3*; P3-2: 5′-GGAATTCCATATGGAAGGTGGGGCCTCTGGGCAACTAC-3′ for *Ray2*, *Rny2*, and *Rny4*); P4: 5′-CCGCTCGAGCTATCACTGGCTGGCCGACAATGCG-3′. A *Nde*I restriction endonuclease cleavage site (underlined sequences) with protective bases was added on the 5′ terminal of P3-1 and P3-2, and an *Xho*I cleavage site on the 5′ terminal of P4. The PCR products were cloned into the bacterial expression vector pET-30a (Novagen, Madison, WI, USA). The recombinant plasmids were transformed into BL21(DE3)pLysS competent cells, and HMW-GSs were expressed in *E. coli* by inducing with 1 mM isopropyl β-d-1-thiogalactopyranoside (IPTG) for 6 h. The overexpressed proteins were isolated from the bacterial cells with an extraction buffer (50% 1-propanol, 1% dithiothreitol (DTT)) at 60 °C for 1 h. The supernatants were mixed with Laemmli buffer (62.5 mM Tris-HCl pH 6.8, 2% SDS, 1.5% DTT, 10% glycerol, 0.002% bromophenol blue), boiled for 10 min, centrifuged, and analyzed by SDS-PAGE.

### 4.6. Sequence Comparison and Phylogenetic Analyses of Cloned HMW-GS Genes

The cloned DNA sequences were assembled, and the ORFs of the HMW-GS genes were translated into amino acid sequences using the DNAMAN software (Version 6.0.3, Lynnon Biosoft, Vaudreuil, QC, Canada). Multiple alignments of the DNA or protein sequences were carried out separately with MEGA6 [37]. In order to investigate the phylogenetic relationships of the new *Roegneria* HMW-GS with previously-characterized *Glu-1* alleles from wheat and related species, the amino acid sequences of the N-terminal with the first three motifs of the central repeat domain and the amino acid sequences of the C-terminal with the last six motifs of the central repeat domain were used to create the ML phylogenetic trees using MEGA6, respectively [37]. A total of 42 HMW-GSs were involved in this phylogenetic analysis, including the six novel *Roegneria* subunits and the other 36 representative subunits from wheat and related species with different genomes. The D-hordein gene (AY268139) from *Hordeum vulgare* was used as the outgroup. The clade support values were estimated using 500 bootstrap pseudoreplicates. The DNA or protein sequences used for multiple alignments and phylogenetic analysis in this research were derived from the NCBI database [30].

## 5. Conclusions

In the present work we cloned six HMW-GS genes from two tetraploid *Roegneria* species, *R. nakaii* and *R. alashanica*. These novel *Roegneria* HMW-GS genes extended and enriched HMW-GS gene families. All of the six *Roegneria* HMW-GSs are not typical x- or y-type subunits and should be classed into an intermediate type inclining towards the y-type, which plausibly represents an evolution status of the *Glu-1* locus. The *Roegneria* HMW-GSs, together with other HMW-GSs from species with St, StY, or StH genome(s) formed an independent clade in phylogenetic trees, varying from the typical x- and y-type clusters. We speculate that the *Glu-1* locus in *R. nakaii* and *R. alashanica* is a very primitive glutenin locus across evolution. In addition, the six *Roegneria* HMW-GS genes were phylogenetically split into two groups, each group included the HMW-GSs from species with St, StY, and StH genomes, demonstrating that the six *Roegneria* HMW-GS genes were from the St genome rather than Y. Nevertheless, the two derived genomes of the two *Roegneria* HMW-GS gene groups have undergone slight differentiation.

## Figures and Tables

**Table 1:** A summary of some information of the six new HMW-GS genes from *R. nakaii* and *R. alashanica*.

Genes	GenBank Accession Number	ORF Size	Signal Peptide	N-terminal		Repetitive Domain		C-terminal		Total	
			Size	Size	Cys	Size	Cys	Size	Cys	Size	Cys
*Rny1*	KP121402	1371 bp	18	102	5	293	0	42	1	455	6
*Rny2*	KP121401	1253 bp	21	105	5	250	0	41	1	415	6
*Rny3*	KP121404	1452 bp	18	102	5	320	0	42	1	482	6
*Rny4*	KP121403	1280 bp	21	105	5	259	0	41	1	424	6
*Ray1*	KP121400	1389 bp	18	102	5	299	0	42	1	461	6
*Ray2*	KP121399	1271 bp	21	105	5	238	0	41	1	403	6

## Supplementary Materials

Supplementary materials can be found at http://www.mdpi.com/1422-0067/17/7/1115/s1.

## References

[1] Payne P.I., Seekings J.A., Worland A.J., Jarvis M.G., Holt L.M. (1987). Allelic variation of glutenin subunits and gliadins and its effect on breadmaking quality in wheat: Analysis of F_5_ progeny from Chinese Spring × Chinese Spring (Hope 1A). J. Cereal Sci..

[2] Shewry P.R., Halford N.G. (2002). Cereal seed storage proteins structures properties and role in grain utilization. J. Exp. Bot..

[3] Payne P.I., Holt L.M., Worland A.J., Law C.N. (1982). Structural and genetical studies on the high-molecular-weight subunits of wheat glutenin: Part 3. Telocentric mapping of the subunit genes on the long arms of the homoeologous group 1 chromosomes. Theor. Appl. Genet..

[4] Payne P.I. (1987). Genetics of wheat storage proteins and the effect of allelic variation on bread-making quality. Annu. Rev. Plant Physiol..

[5] Payne P.I., Corfield K.G., Holt L.M., Blackman J.A. (1981). Correlations between the inheritance of certain high-molecular weight subunits of glutenin and bread-making quality in progenies of six crosses of bread wheat. J. Sci. Food Agric..

[6] Shewry P.R., Lafiandra D., Tamas L., Bekes F., Wrigley C., Bekes F. (2006). Genetic manipulation of gluten structure and function. Gliadin and Glutenin: The Unique Balance of Wheat Quality.

[7] Halford N.G., Forde J., Anderson O.D., Greene F.C., Shewry P.R. (1987). The nucleotide and deduced amino acid sequences of an HMW glutenin subunit gene from chromosome 1B of bread wheat (*Triticum aestivum* L.) and comparison with those of genes from chromosomes 1A and 1D. Theor. Appl. Genet..

[8] Anderson O.D., Greene F.C., Yip R.E., Halford N.G., Shewry P.R., Malpica-Romero J.M. (1989). Nucleotide sequences of the two high weight glutenin genes from the D-genome of a hexaploid bread wheat, *Triticum aestivum* L. cv Cheyenne. Nucleic Acids Res..

[9] Li W., Wan Y., Liu Z., Liu K., Liu X., Li B., Li Z., Zhang X., Dong Y., Wang D. (2004). Molecular characterization of HMW glutenin subunit allele *1Bx14*: Further insights into the evolution of *Glu-B1-1* in wheat and related species. Theor. Appl. Genet..

[10] Pang B.S., Zhang X.Y. (2008). Isolation and molecular characterization of high molecular weight glutenin subunit genes *1Bx13* and *1By16* from hexaploid wheat. J. Integr. Plant Biol..

[11] Shao H., Liu T., Ran C.F., Li L.Q., Yu J., Gao X., Li X.J. (2015). Isolation and molecular characterization of two novel HMW-GS genes from Chinese wheat (*Triticum aestivum* L.) landrace Banjiemang. Genes Genom..

[12] Bustos A.D., Jouve N. (2003). Characterisation and analysis of new HMW-glutenin alleles encoded by the *Glu-R1* locus of *Secale*
*cereale*. Theor. Appl. Genet..

[13] Guo Z.F., Yan Z.H., Wang J.R., Wei Y.M., Zheng Y.L. (2005). Characterization of HMW prolamines and their coding sequences from *Crithopsis delileana*. Hereditas.

[14] Li Z.X., Zhang X.Q., Zhang H.G., Cao S.H., Wang D.W., Hao S.T., Li L.H., Li H.J., Wang X.P. (2008). Isolation and characterization of a novel variant of HMW glutenin subunit gene from the St genome of *Pseudoroegneria stipifolia*. J. Cereal Sci..

[15] Liu S.W., Gao X., Xia G.M. (2008). Characterizing HMW-GS alleles of decaploid *Agropyron elongatum* in relation to evolution and wheat breeding. Theor. Appl. Genet..

[16] Jiang Q.T., Wei Y.M., Lu Z.X., Pu Z.E., Lan X.J., Zheng Y.L. (2010). Structural variation and evolutionary relationship of novel HMW glutenin subunits from *Elymus glaucus*. Hereditas.

[17] Liu S.W., Zhao F., Gao X., Chen F.G., Xia G.M. (2010). A novel high molecular weight glutenin subunit from *Australopyrum retrofractum*. Amino Acids.

[18] Dai S., Pu Z., Liu D., Wei Y., Zheng Y., Hu X., Yan Z. (2013). Characterization of novel HMW-GS in two diploid species of *Eremopyrum*. Gene.

[19] Cao S., Li Z., Gong C., Xu H., Yang R., Hao S., Wang X., Wang D., Zhang X. (2014). Identification and characterization of high-molecular-weight glutenin subunits from *Agropyron intermedium*. PLoS ONE.

[20] Kong L., Liang Y., Qin L., Sun L., Xia G., Liu S. (2014). Characterization of high molecular weight glutenin subunit genes from the Ns genome of *Psathyrostachys juncea*. Dev. Genes Evol..

[21] Sun Y., Pu Z., Dai S., Pu X., Liu D., Wu B., Lan X., Wei Y., Zheng Y., Yan Z. (2014). Characterization of y-type high-molecular-weight glutenins in tetraploid species of *Leymus*. Dev. Genes Evol..

[22] Wang S.B., Han H.N., Liang Y., Sun L., Xia G.M., Liu S.W. (2014). Isolation and characterization of novel *Glu-St1* alleles from *Pseudoroegneria spicata* and *Pd. strigosa*. Genetica.

[23] Zhu G., Wang S., Zhen S., Shen X., Prodanovic S., Yan Y. (2015). Molecular characterization and phylogenetic analysis of unusual x-type HMW glutenin subunits from 1S^L^ genome of *Aegilops longissima*. Genetika.

[24] Shewry P.R., Halford N.G., Tatham A.S., Popineau Y., Lafiandra D., Belton P.S. (2003). The high molecular weight subunits of wheat glutenin and their role in determining wheat processing properties. Adv. Food Nutr. Res..

[25] Baum B.R., Yen C., Yang J.L. (1991). *Roegneria*: Its generic limits and justification for its recognition. Can. J. Bot..

[26] Yang J.L., Baum B.R., Yen C. (2008). A revision of the genus *Roegneria* C. Koch (Triticeae: Poaceae). J. Sichuan Agric. Univ..

[27] Dong Y.S., Zhou R.H., Xu S.J., Li L.H., Cauderon Y., Wang R.R.-C. (1992). Desirable characteristics in perennial Triticeae collected in China for wheat improvement. Hereditas.

[28] Wan Y.F., Yen C., Yang J.L., Liu F.Q. (1987). Evaluation of *Roegneria* for resistance to head scab caused by *Fusarium graminearum* Schwabe. Genet. Resour. Crop Evol..

[29] Wang X.E., Chen P.D., Liu D.J., Zhang P., Zhou B., Friebe B., Gill B.S. (2001). Molecular cytogenetic characterization of *Roegneria ciliaris* chromosome additions in common wheat. Theor. Appl. Genet..

[30] National Center for Biotechnology Information (NCBI) GenBank. https://www.ncbi.nlm.nih.gov/genbank/.

[31] Shewry P.R., Napier J.A., Tatham A.S. (1995). Seed storage proteins: Structures and biosynthesis. Plant Cell.

[32] Liu S.T., Zhu X.L., Tan Y., Liu S.W. (2012). Isolation and characterization of *Glu-1* genes from the St genome of *Pseudoroegneria libanotica*. Gene.

[33] Li S.F., Zhou H.B., Li L.H., Wang X.P., Zhang X.Q. (2007). Isolation and characterization of high-molecular-weight glutenin subunit genes in *Agropyron cristatum*. Acta Agric. Sin..

[34] Zhang L., Chen Q., Su M., Yan B., Zhang X., Jiao Z. (2016). High-molecular-weight glutenin subunit-deficient mutants induced by ion beam and the effects of *Glu-1* loci deletion on wheat quality properties. J. Sci. Food Agric..

[35] Denery-Papini S., Popineau Y., Quillien L., van Regenmortel M.H.V. (1996). Specificity of antisera raised against synthetic peptide fragments of high Mr glutenin subunits. J. Cereal Sci..

[36] Webb D.M., Knapp S.J. (1990). DNA extraction from a previously recalcitrant plant genus. Plant Mol. Biol. Rep..

[37] Tamura K., Stecher G., Peterson D., Filipski A., Kumar S. (2013). MEGA6: Molecular evolutionary genetics analysis version 6.0. Mol. Biol. Evol..

