# Supplementary material for: Structural Characterization and Evolutionary Relationship of High-Molecular-Weight Glutenin Subunit Genes in Roegneria nakaii and Roegneria alashanica
Source: Int J Mol Sci. 2016 Jul 19;17(7):1115. doi: 10.3390/ijms17071115 (PMC4964490; doi:10.3390/ijms17071115)
Supplement: Supplementary file 1 [file ijms-17-01115-s001.pdf]

Supplementary Materials: Structural Characterization and Evolutionary Relationship of High-Molecular-Weight Glutenin Subunit Genes in *Roegneria nakaii* and *Roegneria alashanica*

Lujun Zhang, Zhixin Li, Renchun Fan, Bo Wei and Xiangqi Zhang

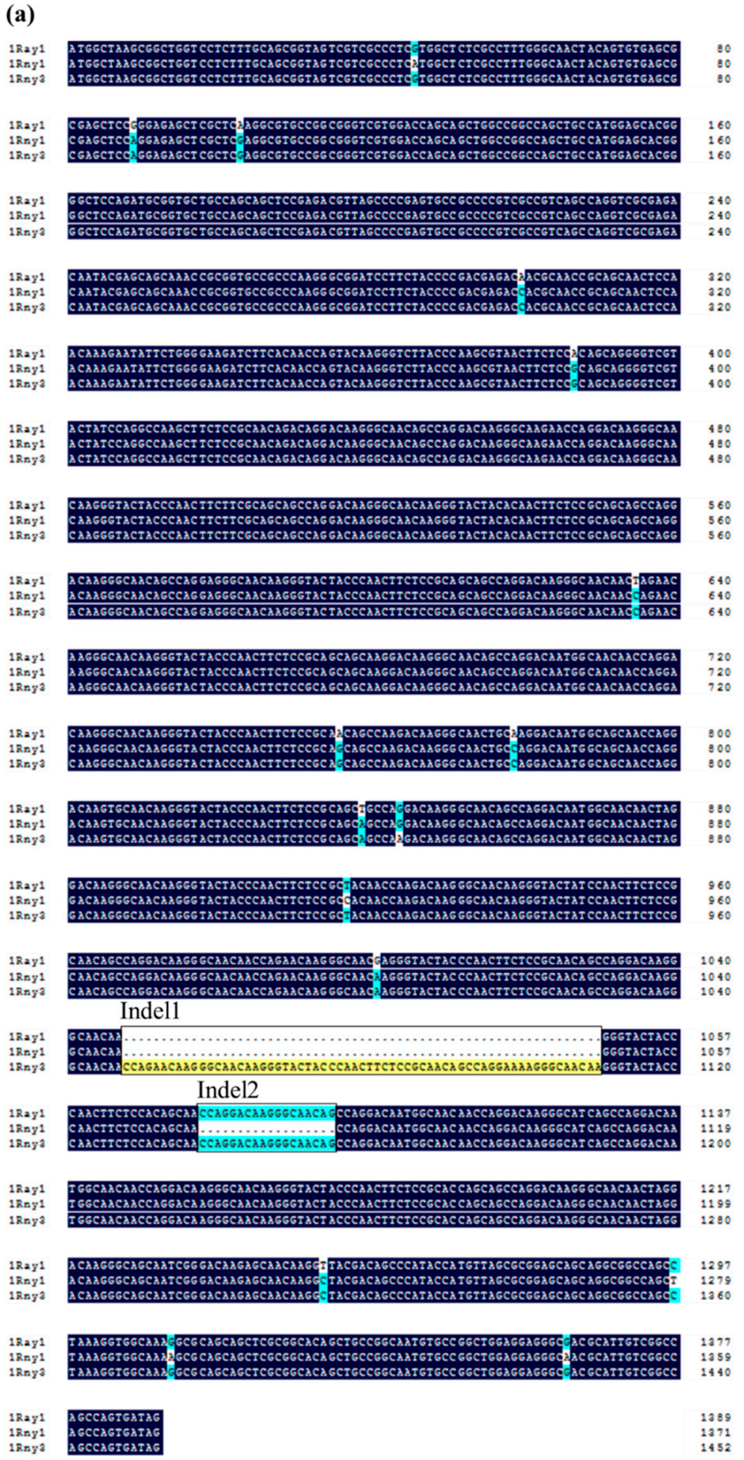

Figure S1. *Cont.*

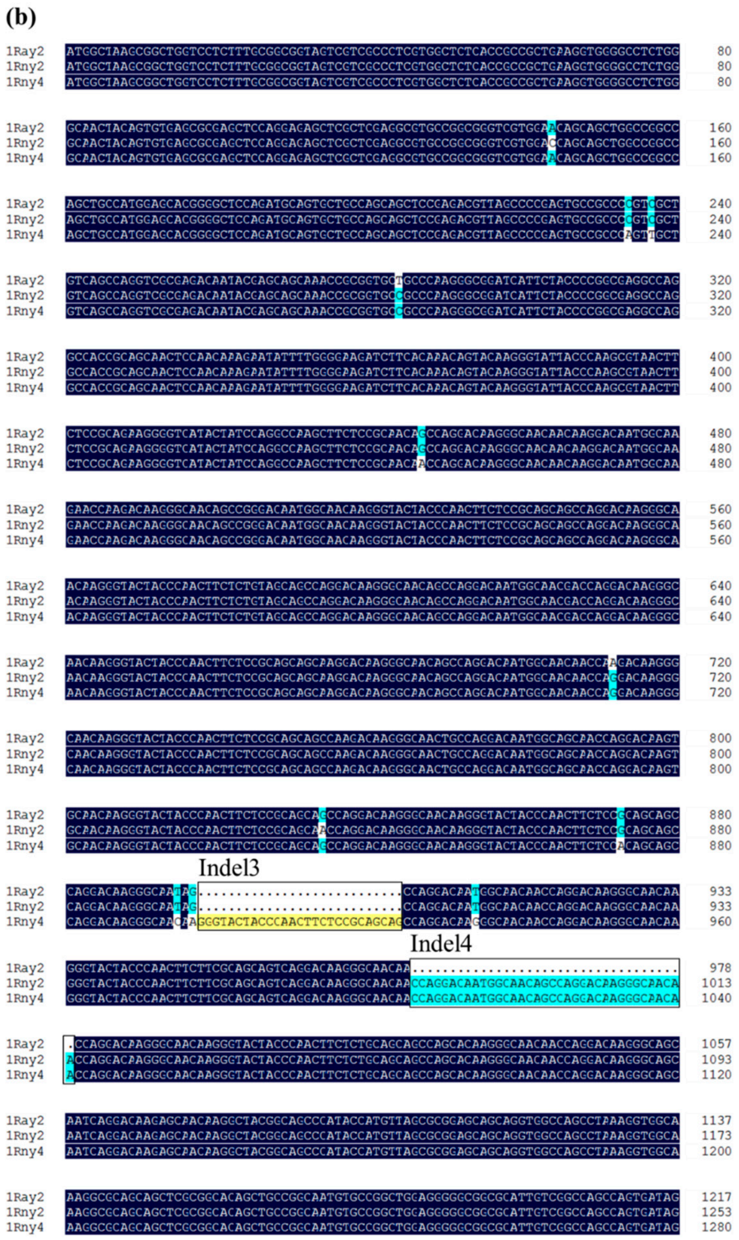

Figure S1. Comparison of the coding region sequences of *Roegneria* high-molecular-weight glutenin subunit (HMW-GS) genes *Rny1*, *Rny3* and *Ray1* (**a**), and *Rny2*, *Rny4* and *Ray2* (**b**). Indels are boxed.
